# An introduction to heart rate variability: methodological considerations and clinical applications

**DOI:** 10.3389/fphys.2015.00055

**Published:** 2015-02-25

**Authors:** George E. Billman, Heikki V. Huikuri, Jerzy Sacha, Karin Trimmel

**Affiliations:** ^1^Department of Physiology and Cell Biology, The Ohio State UniversityColumbus, OH, USA; ^2^Division of Cardiology, Department of Internal Medicine, Institute of Clinical Medicine, University of OuluOulu, Finland; ^3^Department of Cardiology, Regional Medical CenterOpole, Poland; ^4^Department of Neurology, Medical University of ViennaVienna, Austria

**Keywords:** heart rate variability, heart rate, heart rate dynamics, autonomic nervous system, risk assessment, cardiovascular disease

Heart rate variability (HRV), the beat-to-beat variation in either heart rate or the duration of the R-R interval, has become a popular clinical and investigational tool (Task Force of the European Society of Cardiology and the North American Society of Pacing and Electrophysiology, 1996; Billman, 2011). Indeed, the term “heart rate variability” yields nearly 18,000 “hits” when placed in the pubmed search engine. These temporal fluctuations in heart rate exhibit a marked synchrony with respiration (increasing during inspiration and decreasing during expiration—the so called respiratory sinus arrhythmia) and are widely believed to reflect changes in cardiac autonomic regulation (Billman, 2011). Although the exact contributions of the parasympathetic and the sympathetic divisions of the autonomic nervous system to this variability are controversial and remain the subject of active investigation and debate, a number of time and frequency domain techniques have been developed to provide insight into cardiac autonomic regulation in both health and disease (Billman, 2011). It is the purpose of this book to provide a comprehensive assessment of the strengths and limitations of HRV techniques. Particular emphasis will be placed on the application of HRV techniques in the clinic and on the interaction between prevailing heart rate and HRV. This book contains both state-of-the art review and original research articles that have been grouped into two main sections: Methodological Considerations and Clinical Application. A brief summary of the chapters contained in each section follows below.

## Methodological considerations

The opening section provides a historical overview of the evolution in the concept of heart rate variability (Billman, 2011) and then describes time domain, frequency domain, and non-linear dynamic analysis techniques (and their limitations) that are commonly used to measure heart rate variability. Heathers (2014) and Billman (2013a) describe methodological issues in the analysis of short-term frequency-domain HRV such as the LF band, normalized units, or the LF/HF ratio as well as the influence of external factors on HRV data. These reviews provide substantial information on mathematical concerns in HRV analysis and on the interpretation of the underlying physiological background of HRV power and highlight the necessity of methodological improvement in HRV measurement. Peltola (2012) evaluates the methods used to edit R-R interval time series and how this editing can influence the results obtained by the HRV analysis. The effects of prevailing HR on HRV are further evaluated in series of review and original research articles.

It is not widely appreciated that HRV is significantly associated with average heart rate (HR) and that, as a consequence, HRV actually provides information on two quantities; i.e., HR and its variability (Sacha, 2014a,c). Sacha (2013, 2014b) demonstrate that interpretation of HRV data is further complicated by the inverse non-linear relationship between HR and R–R interval. Owing to this inverse (mathematical) relationship, the same fluctuations of HR yield higher R-R interval changes for the slow than for the fast average HR, and therefore the standard analysis of heart rate variability may be mathematically biased (Sacha and Pluta, 2008). Thus, one must calculate HRV normalized to HR in order to differentiate between physiologically and mathematically mediated changes in HRV (Sacha, 2013). This normalization is particularly important if one compares HRV between the patients with different average HRs or during interventions that change HR. The effect of these normalization procedures are explored further in a series of original research articles.

For example, the effects of HR on the HRV response to different autonomic interventions were examined using a canine model (Billman, 2013b). Maneuvers that accelerated HR (e.g., submaximal exercise) caused a decrease in HRV even after normalization for the HR changes while interventions that slowed down HR yielded mixed results (e.g., baroreceptor reflex activation provoked an increase in HRV even after normalization for reflexively mediated reductions in HR, while beta-adrenergic receptor antagonists reduced rather than increased HRV after normalization for the drug-induced HR reductions) (Billman, 2013b). In a review article, Billman (2013a) further demonstrated that, among other factors, both heart rate and mathematical considerations profoundly influence the LF/HF ratio such that it is not possible to determine the physiological basis for this widely use index (Billman, 2013a). He concluded that the preponderance of evidence confirms that the LF/HF ratio cannot accurately quantify cardiac “sympatho-vagal balance” either in health or disease (Billman, 2013a).

In another article, Grant et al. demonstrate (by employment of the normalization method) that HR is a better indicator of higher fitness than HRV; i.e., an association between HRV indices and maximal oxygen intake (VO_2_max) exists mainly due to the relationship between HR and VO_2_max (Grant et al., 2013). On the other hand, the same normalization method enabled Carter et al. to show that an increase in HRV following dengue viral infection does not result from the accompanying reduction in HR, but reflects a real improvement in cardiac autonomic nervous control (Carter et al., 2014). Finally, Pradhapan et al. (2014) examined the impact of HR on HRV on the results of exercise stress testing and found that HR immediately before exercise was not a risk factor of death, and the removal of its influence improved the HRV predictive power. Conversely, HR during the recovery phase was a significant mortality predictor, and the enhancement of its impact (by using the method of Sacha et al., 2013) increased the HRV predictive ability (Pradhapan et al., 2014). These examples clearly show that it is very important to establish to what extent HRV changes associated with simultaneous HR alterations are physiologically and mathematically determined. Unraveling this remarkable interplay between HRV and HR may yield valuable prognostic information (Sacha, 2014b). Further studies are needed to determine which of the two, i.e. HR or HRV, provides better predictive performance for a given population and outcome as well as to what modifications of the HRV/HR relationship increase the prognostic power of HRV (Sacha, 2014b).

## Clinical applications

HRV analysis has become an increasing important diagnostic tool in cardiology. For example, Lombardi and Stein (2011) review the relationship between HRV and heart rate turbulence (HRT, baroreceptor reflex mediated short-term oscillations in the heart period that occur after spontaneous ventricular arrhythmias) and “sympatho-vagal” balance while Zuern et al. (2011) and Huikuri and Stein (2012) evaluate HRV and HRT as tools for risk assessment in patients recovering from myocardial infarction. Non-linear indices of HRV are evaluated by Perkiömäki (2011) and Glass et al. (2011). Perkiömäki (2011) reports that novel HRV indices that quantify the non-linear dynamics of HR may have a greater prognostic value to identify patients with the greatest risk for adverse cardiovascular events than do conventional HRV indices, while Glass et al. (2011) analyzed the dynamic properties of premature ventricular complexes to reveal the underlying mechanisms responsible for these arrhythmias.

In a similar fashion, Papaioannou et al. (2013) investigated the association between changes in HRV and the inflammatory response in patients with cardiovascular diseases by assessing the relationship of inflammatory biomarkers such as CRP, TNF-a, IL6, or white blood cell count with different parameters of HRV. Bravi et al. (2013) further explored the different changes in HRV produced by physiological and pathological stress. Datasets of healthy subjects performing physiological exercise (physiological stress) were compared to those of patients who developed sepsis after a bone marrow transplant (pathological stress), showing similar responses during both conditions, however, with subtle differences. In another chapter, Jelinek et al. (2013) evaluated cardiac rehabilitation (CR) outcomes following a 6-week program of percutaneous coronary revascularization (PCI) and coronary artery bypass graft (CABG) patients by the analysis of HRV variables and comparing changes in the 6-min-walk-test and peak VO_2_. It was shown that CR significantly improved exercise capacity and positively affected HRV changes especially in the CABG group. Hinojosa-Laborde et al. (2011) investigated whether any HRV index could accurately distinguish between individuals with high and low tolerances to simulated hemorrhage (i.e., lower body negative pressure). They report that, although a few HRV indices could accurately differentiate between low and high tolerance subjects when considered as group (i.e., difference in group means), a given individual's HRV value provided a poor indicator of tolerance to hypovolemia. Finally, Tobaldini et al. (2013) reviewed linear and non-linear analyses of HRV to assess autonomic changes during sleep under physiological as well as pathological conditions such as sleep-related breathing disorders, insomnia, or epilepsy/sudden unexplained death in epilepsy (SUDEP).

Thus, by understanding both the strengths and limitations of the various techniques used to quantify heart rate variability, the authors hope that this brief monograph will provide sufficient knowledge so that these indices can be used appropriately in the clinic not only to identify high risk patients but also to aid in the development of more effective therapies to treat the diseases that elicited the HRV changes.

### Conflict of interest statement

The authors declare that the research was conducted in the absence of any commercial or financial relationships that could be construed as a potential conflict of interest.
